# HSP47 Increases the Expression of Type I Collagen in Fibroblasts through IRE1α Activation, XBP1 Splicing, and Nuclear Translocation of β-Catenin

**DOI:** 10.3390/cells13060527

**Published:** 2024-03-17

**Authors:** So Young Ham, Min Ju Pyo, Moonkyung Kang, Yeon-Soo Kim, Dong Hun Lee, Jin Ho Chung, Seung-Taek Lee

**Affiliations:** 1 Department of Biochemistry, College of Life Science and Biotechnology, Yonsei University, Seoul 03722, Republic of Korea; soyoung@yonsei.ac.kr (S.Y.H.); vyalswn2525@naver.com (M.J.P.); 2 R&D Center, artiCure Inc., Daejeon 34134, Republic of Korea; 3 Graduate School of New Drug Discovery and Development, Chungnam National University, Daejeon 34134, Republic of Korea; 4 Department of Dermatology, Seoul National University College of Medicine, Seoul 03080, Republic of Korea; ivymed27@snu.ac.kr (D.H.L.); jhchung@snu.ac.kr (J.H.C.); 5 Laboratory of Cutaneous Aging Research, Biomedical Research Institute, Seoul National University Hospital, Seoul 03080, Republic of Korea; 6 Institute of Human-Environment Interface Biology, Seoul National University, Seoul 03080, Republic of Korea; 7 Institute on Aging, Seoul National University, Seoul 03080, Republic of Korea

**Keywords:** HSP47, fibroblast, type I collagen, extracellular matrix, skin aging

## Abstract

Heat shock protein 47 (HSP47), also known as *SERPINH1*, functions as a collagen-specific molecular chaperone protein essential for the formation and stabilization of the collagen triple helix. Here, we delved into the regulatory pathways governed by HSP47, shedding light on collagen homeostasis. Our investigation revealed a significant reduction in *HSP47* mRNA levels in the skin tissue of older mice as compared to their younger counterparts. The augmented expression of HSP47 employing lentivirus infection in fibroblasts resulted in an increased secretion of type I collagen. Intriguingly, the elevated expression of HSP47 in fibroblasts correlated with increased protein and mRNA levels of type I collagen. The exposure of fibroblasts to IRE1α RNase inhibitors resulted in the reduced manifestation of HSP47-induced type I collagen secretion and expression. Notably, HSP47-overexpressing fibroblasts exhibited increased *XBP1* mRNA splicing. The overexpression of HSP47 or spliced XBP1 facilitated the nuclear translocation of β-catenin and transactivated a reporter harboring TCF binding sites on the promoter. Furthermore, the overexpression of HSP47 or spliced XBP1 or the augmentation of nuclear β-catenin through Wnt3a induced the expression of type I collagen. Our findings substantiate that HSP47 enhances type I collagen expression and secretion in fibroblasts by orchestrating a mechanism that involves an increase in nuclear β-catenin through IRE1α activation and XBP1 splicing. This study therefore presents potential avenues for an anti-skin-aging strategy targeting HSP47-mediated processes.

## 1. Introduction

Collagen is the most abundant fibrous protein in the extracellular matrix (ECM) and provides mechanical strength, resiliency, and elasticity to skin [1,2,3]. The skin contains various types of collagens, but type I collagen is the most abundant, making up about 80–90% of the collagen content [4,5]. Alterations in the organization and structure of type I collagen are a hallmark of chronologically aged human skin. Over the aging process in skin, type I collagen becomes increasingly fragmented. The fragmented type I collagen decreases the overall strength of the skin and promotes the formation of wrinkles [3].

Heat shock protein 47 (HSP47), encoded by the *SERPINH1* gene, is a molecular chaperone resident in the endoplasmic reticulum (ER) that plays an important role in the correct folding and assembly of procollagen [6]. Procollagen is inherently unstable at physiological temperature and is prone to intracellular degradation. HSP47 binds to the Gly-Xaa-Arg motif of the triple-helical region of procollagen in the ER via hydrophobic and hydrophilic interactions. In HSP47 KO cells, the secretion of type I and IV collagens was delayed compared to HSP47 WT cells [6,7,8]. Type I collagen was highly accumulated in the ER of HSP47 KO cells, whereas was mainly in the Golgi apparatus in HSP47 WT cells [8]. The deficiency of HSP47 did not affect the secretion of other ECM proteins such as fibronectin and laminin, underscoring the specificity of HSP47 in modulating procollagen maturation [9]. In HSP47 KO cells, procollagens are not correctly folded in the triple-helical form and form detergent-insoluble aggregates in the ER. These aggregated procollagens are in part eliminated through autophagy [10]. Although some of type I collagens can be secreted from HSP47 KO cells, they exhibit increased susceptibility to trypsin/chymotrypsin digestion [7,11]. Consequently, HSP47 provides a quality control mechanism for correct helical folding and assembly by effectively chaperoning the formation of the triple helix while simultaneously preventing premature aggregation of the helices [12,13].

In addition to its role in collagen folding and assembly, HSP47 is known to play a role in unfolded protein response (UPR) [14,15]. Three major UPR sensors, inositol-requiring enzyme 1 (IRE1), protein kinase RNA-like endoplasmic reticulum kinase (PERK), and activating transcription factor 6 (ATF6), are known [16,17]. Among them, IRE1 is capable of alleviating ER stress by two distinct routes: the splicing of X-box binding protein 1 (*XBP1*) mRNA and regulated IRE1-dependent decay (RIDD) [18]. Under ER stress, increased ER protein load and the presence of unfolded proteins lead to the dissociation of the ER chaperone binding immunoglobulin protein (BiP) from IRE1. The free IRE1 then dimerizes and autophosphorylates, which leads to activation of the IRE1 RNase domain in the cytosol [19]. The activated IRE1 RNase is involved in splicing of the *XBP1* mRNA, resulting in a frameshift that allows translation of the active XBP1-spliced (XBP1-s) transcription factor [20,21]. XBP1-s enhances ER proteostasis through the increased expression of stress-responsive genes, including protein folding chaperones, such as BiP and HSP47, and ER-associated degradation components, such as SEL1L and HRD1 [22,23]. Activated IRE1 RNase also promotes the degradation of mRNAs encoding ER-localized proteins through a process known as RIDD, which often leads to apoptosis through the downregulation of mRNAs encoding the proteins important for protein folding such as GRP78 [24,25]. Interestingly, HSP47, which is a target gene of the activated IRE1, is known to facilitate the IRE1 activation by binding to the ER luminal domain of IRE1, reducing the binding of BiP [26].

During skin aging, both the epidermis and dermis become thinner, which is due to the reduction in fibroblasts and extracellular collagens in the dermis, as well as a slower turnover rate of the epidermis, which leads to lower skin barrier function [27]. Although HSP47 is well known to regulate the folding and assembly of collagens, a recent study showed that HSP47 has a characteristic expression pattern in cells and tissues that correlates closely with the expression of various types of collagens [9,28]. This correlation suggests a potential regulatory role of HSP47 in the expression of collagens. Consequently, HSP47 emerges as a significant target with the potential to modulate skin thinning during aging by influencing collagen levels in the skin. To explore this possibility, we conducted an analysis of HSP47 expression in the skin tissues of young and old mice. Additionally, we investigated changes in the secretion and expression of type I collagen by HSP47 overexpression in human foreskin fibroblasts. Furthermore, we analyzed possible molecular mechanisms underlying how HSP47 induces changes in collagen biosynthesis in fibroblasts. Collectively, we evaluated the significance of HSP47 as a crucial target for controlling collagen homeostasis and mitigating the effects of skin aging.

## 2. Materials and Methods

### 2.1. Antibodies and Reagents

The anti-pro-collagen α1(I) N-propeptide (pN-Col1Iα1) antibody was described previously [29]. Anti-HSP47 antibody was obtained from Proteintech (Chicago, IL, USA). Anti-XBP1 antibody was purchased from Abcam (Cambridge, UK). Anti-β-catenin antibody was purchased from Cell Signaling Technology (Danvers, MA, USA). Anti-Lamin A/C antibody was purchased from Santa Cruz Biotechnology (Dallas, TX, USA). Anti-GAPDH antibody was purchased from AbClone (Seoul, Republic of Korea). Horseradish peroxidase-conjugated goat anti-mouse IgG and goat anti-rabbit IgG were obtained from KOMA Biotech (Seoul, Republic of Korea). STF083010 and 4µ8C were purchased from Selleck Chemicals (Houston, TX, USA).

### 2.2. Acquisition of Mouse Skin Tissues

Dorsal skin tissues were procured from female albino hairless mice (Skh-1; Orient Bio, Seoul, Republic of Korea), aged 3 months (young) and 24 months (old). The skin tissues were frozen in liquid nitrogen and stored at a temperature of −70 °C. Prior approval was obtained from the Institutional Animal Care and Use Committee for all procedures involving mice as subjects.

### 2.3. RNA Isolation and Reverse Transcription-Polymerase Chain Reaction (RT-PCR) Analysis

Total RNA was extracted from mouse skin tissues and foreskin fibroblasts using TRIZOL (Invitrogen, Carlsbad, CA, USA) in accordance with a previously described procedure [30], with minor modifications. cDNA was produced by utilizing oligo(dT)_15_ primers and AMV RTase (Promega, Madison, WI, USA) to reverse transcribe the total RNA. For conventional PCR amplification, a final volume of 10 μL was utilized. This volume contained the following components: 0.2 mM dNTPs, 1× Taq PCR buffer, 50 U/mL of Taq polymerase, 1 pM of 5′-primer and 3′-primer for each reaction (Table S1), and cDNAs synthesized from 0.1 µg total RNA. PCR was conducted for 16–30 cycles. Each cycle consisted of denaturation at 94 °C for 30 s, annealing at an annealing temperature (Table S1) for 60 s, and extension at 72 °C for 30 s. PCR product detection was accomplished via 5% PAGE and ethidium bromide staining. Quantitation of the PCR products detected in PAGE was carried out using ImageJ software. The expression levels of the target genes were standardized to GAPDH in the respective samples. For real-time quantitative PCR, the annealing temperature was identical to that of conventional PCR. To detect real-time PCR products, the QuantiTect SYBR Green PCR kit (Qiagen, Hilden, Germany) and the QuantStudio 3 Real-Time PCR system (Applied Biosystems, Foster City, CA, USA) were used.

### 2.4. Preparation of Skin Tissue Extracts and Western Blot Analysis

The frozen skin tissues were crushed into a fine powder using liquid nitrogen. The powder was then homogenized with the addition of a radioimmunoprecipitation assay (RIPA) lysis buffer (50 mM Tris-HCl, pH 7.4, 150 mM NaCl, 1% NP-40, 0.5% sodium deoxycholate, 0.1% sodium dodecyl sulfate [SDS]), including a protease inhibitor cocktail (Calbiochem, San Diego, MA, USA). The homogenized skin tissues underwent sonication and were subsequently centrifuged at 14,000× *g* for 20 min at 4 °C to extract proteins in the supernatant. The protein extracts from tissues were combined with 5× SDS sample buffer (250 mM Tris-HCl, pH 6.8, 10% SDS, 0.5% bromophenol blue, and 50% glycerol) and boiled in the presence of 100 mM β-mercaptoethanol. The mixture was then used in SDS-PAGE and Western blotting. The proteins in SDS-PAGE were transferred to a PVDF membrane (Millipore, Billerica, MA, USA). The blots were blocked with a 5% skim milk solution and subsequently exposed to primary and secondary antibodies. The immunoreactive bands were detected using the West-Q PICO Dura ECL solution (GenDepot, Barker, TX, USA), Immobilon Western Chemiluminescent HRP Substrate (Millipore), and Amersham ImageQuant 800 (Cytiva, Marlborough, MA, USA). The protein bands detected by Western blotting were quantified using ImageJ software.

### 2.5. Cell Culture

Human primary foreskin fibroblasts were obtained from Welgene Inc. (Gyeongsan, Republic of Korea). Foreskin fibroblasts were cultured in complete DMEM (Hyclone, South Logan, UT, USA) with 10% FBS (Gibco/Thermo Fisher Scientific, Waltham, MA, USA), while HEK293T cells were cultured in complete DMEM with 10% bovine serum (BS; Gibco/Thermo Fisher Scientific), along with 100 U/mL of penicillin and 100 μg/mL streptomycin. The cells were maintained at 37 °C in an atmosphere consisting of 5% CO_2_ and 95% air.

### 2.6. Construction of Lentiviral Transfer Vectors Harboring Human HSP47 and XBP1-s cDNAs

The human HSP47 expression vector pOTB7-hHSP47(hSERPINH1), encoding a full-length human HSP47 cDNA (GeneBank NM_001235), was obtained from the Korea Human Gene Bank (Daejeon, Republic of Korea). To construct the pcDNA3.1-HSP47 vector, the full-length HSP47 cDNA fragment from pOTB7-hHSP47 was digested with NheI and KpnI and then ligated into the NheI-KpnI sites of pcDNA3.1(+) (Invitrogen). The human XBP1-s expression vector pcDNA3.1-XBP1-s, encoding a full-length human XBP1-s cDNA (GeneBank NM_001079539), was generously provided by Prof. J. B. Yoon at Yonsei University, Republic of Korea. The transfer of a cDNA fragment from the pcDNA3.1 vector to the pHRST-IRES-eGFP lentiviral transfer vector was performed as described previously [31]. The resulting pHRST-HSP47-IRES-eGFP and pHRST-XBP1-s-IRES-eGFP vectors were sequenced to ensure the absence of PCR errors.

### 2.7. Production and Infection of Lentiviruses

HEK293T cells were co-transfected for 16 h at a ratio of 6:3:2 for the transfer vector, packaging vector (psPAX2), and envelope vector (pMD2.G) (Addgene, Cambridge, MA, USA). The transfer vector was pHRST-IRES-eGFP, pHRST-HSP47-IRES-eGFP, or pHRST-XBP1-s-IRES-eGFP. The medium was substituted with fresh complete medium containing 20 mM HEPES, pH 7.9, and the cells were harvested after a 48-h incubation period, as described previously [32]. The lentivirus-containing conditioned medium was stored at −70 °C. The lentiviruses were introduced into fibroblasts for 16 h using a 2:1 mix of virus-containing medium and fresh medium with 8 µg/mL polybrene.

### 2.8. Preparation of Conditioned Media and Cell Lysates and Western Blot Analysis

Following lentiviral infection, fibroblasts were incubated for 24 h in complete medium before being rinsed twice with PBS. Without further specification, fibroblasts were incubated in serum-free medium for 24 h. If necessary, IRE1α RNase inhibitors, STF083010 (40 μM) or 4μ8C (50 μM), were added to the serum-free medium. For the analysis of type I collagen secretion, the conditioned media were collected by centrifugation at 2000× *g* for 5 min. For the analysis of protein levels in cell lysates, cells were lysed with 1× SDS sample buffer as described previously [33,34]. For nuclear and cytosolic fractionation, cells were lysed with 0.1% NP-40 on ice and centrifuged at 12,000× *g* for 30 s, as described previously [35]. Protein samples were boiled for 3 min in the presence of 100 mM β-mercaptoethanol. The Western blotting procedure for conditioned media and cell lysates was identical to that used for tissue extracts, as described above.

### 2.9. Production and Treatment of a Wnt3a-Containing Conditioned Medium

L-Wnt3a cells, which secrete Wnt3a, and control L cells (kindly provided by Prof. Kang-Yell Choi, Yonsei University, Republic of Korea) were cultured in complete medium supplemented with 10% FBS. Subconfluent cells were incubated with fresh complete medium, and the conditioned medium was harvested after 4 d of incubation. The conditioned medium was stored at −20 °C after being centrifuged at 2000× *g* for 5 min at 4 °C. Fibroblasts were treated with an 8:2 ratio of conditioned medium to fresh medium. Complete fresh medium was used for luciferase analysis, and serum-free fresh medium was used for Western blot analysis.

### 2.10. Dual-Luciferase Reporter Assay

After a 16 h infection with lentiviruses, foreskin fibroblasts (5 × 10^4^ cells/well) were seeded into 24-well plates containing complete medium. The cells were rinsed twice with PBS prior to transfection and replaced with 450 μL of Opti-MEM (Gibco/Thermo Fisher Scientific). The procedure for transfecting reporter constructs into foreskin fibroblasts was carried out using Lipofectamine LTX (Thermo Fisher Scientific), as previously described [36] with slight modifications. pGL3-OT or pGL3-OF plasmid (0.5 μg each) encoding firefly luciferase with the wild-type or mutant TCF promoter, respectively, and pRL-TK (0.05 μg, Promega) encoding *Renilla* luciferase driven by the herpes simplex virus thymidine kinase promoter in 25 µL Opti-MEM were mixed with Lipofectamine LTX (0.75 µL, Invitrogen) and PLUS reagent (0.25 µL, Invitrogen) in 25 µL Opti-MEM. The mixture was incubated for 20 min at room temperature. After being treated with this mixture for 4 h, the cells were incubated for 20 h in complete medium. The dual-luciferase reporter assay system (Promega) was utilized to quantify luciferase activity. The firefly luciferase activity in transfected cells was normalized to *Renilla* luciferase activity.

## 3. Results

### 3.1. Expression of HSP47 Is Downregulated in Old Mouse Skin Tissues

**Figure 1:**
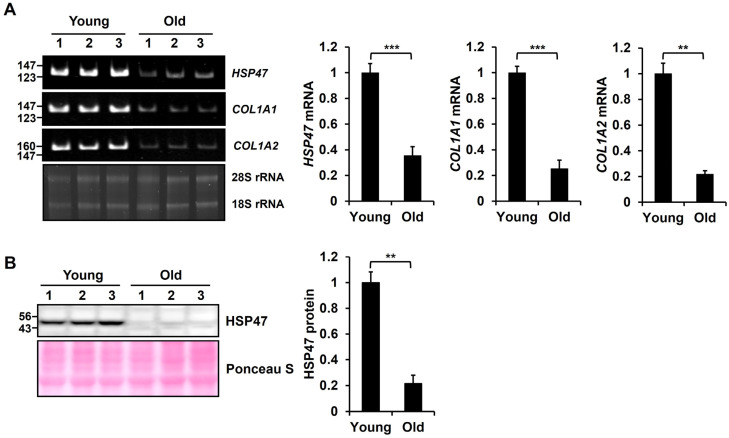
Analysis of HSP47 expression in young and old mouse skin tissues. (**A**) mRNA levels of *HSP47*, *COL1A1*, and *COL1A2* genes in young and old mouse skin tissues were analyzed by RT-PCR. The left panel displays images of RT-PCR products on polyacrylamide gels and total RNA (500 ng) on an agarose gel for the detection of rRNA levels. Relative mRNA levels were normalized using 28S and 18S rRNA levels. The right panel shows a graphical presentation of *HSP47, COL1A1,* and *COL1A2* mRNA levels in mouse skin tissues. (**B**) The protein level of HSP47 in young and old mouse skin tissues was analyzed by Western blotting. Samples were subjected to 10% SDS-PAGE. Protein levels were normalized by Ponceau S-stained protein amounts. Values relative to that in skin tissues of young mice (n = 3) are shown as means ± SD. ** *p* < 0.01, *** *p* < 0.001 vs. Young.

To analyze the change in expression in *HSP47* mRNA levels in mouse skin tissues during aging, we determined *HSP47* mRNA levels in the skin tissues of young (3 months old) and aged (24 months old) mice. *HSP47* mRNA levels exhibited a significant decrease in the skin tissues of aged mice compared with young mice, as evidenced by conventional RT-PCR (Figure 1A). According to the RT-PCR results, it was determined that *HSP47* significantly decreased to 35% ± 6.9% in old mouse tissues compared to young mouse tissues. As reported, *COL1A1* and *COL1A2* mRNA levels also decreased in old mouse skin tissues compared to young mouse skin tissues. Additionally, we conducted an analysis using the Western blot method to assess changes in the protein levels of HSP47 in mouse skin tissues during aging. Similar to the mRNA levels, the protein levels of HSP47 exhibited a significant decrease to 21% ± 6.3% in old mice compared to young mice (Figure 1B). Therefore, we hypothesized that the reduction in the HSP47 protein plays a role in the decrease in type I collagen in aged skin.

### 3.2. HSP47 Overexpression Enhances the Secretion and Expression of Type I Collagen in Human Fibroblasts

**Figure 2:**
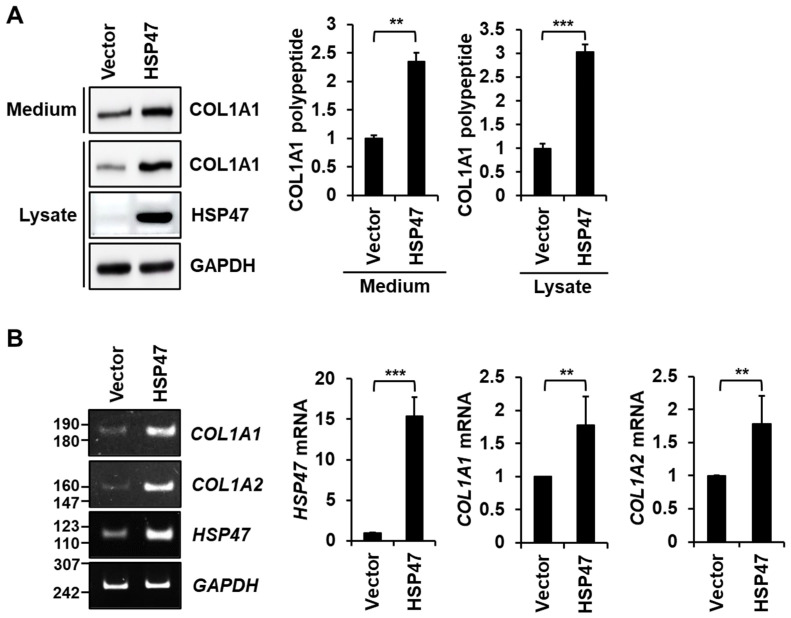
Effect of HSP47 expression on the secretion and expression of type I collagen in human fibroblasts. Human foreskin fibroblasts were infected with vector or HSP47 lentivirus. (**A**) After viral infection, the cells were incubated in complete medium for 24 h and then with serum-free DMEM for an additional 24 h. Media and cell lysate samples were subjected to 9% SDS-PAGE and Western blotting. Protein levels were normalized by GAPDH levels. (**B**) mRNA levels of the *HSP47*, *COL1A1*, and *COL1A2* genes were analyzed by conventional and quantitative RT-PCR at 24 h of incubation in complete medium after infection. Relative mRNA levels were normalized by *GAPDH* mRNA levels. Each value represents the mean ± SD of three independent experiments. ** *p* < 0.01, *** *p* < 0.001 vs. vector virus infection.

We investigated the effect of HSP47 on the secretion of type I collagen in human foreskin fibroblasts. To achieve HSP47 overexpression in fibroblasts, a lentiviral expression system co-expressing GFP was employed. Following the infection of fibroblasts with HSP47 lentivirus and a control lentivirus containing the vector, comparable levels of GFP expression were observed in the fibroblasts (Figure S1), indicating that their infection efficiencies were similar. The overexpression of HSP47 resulted in a significant increase to 230% ± 14.7% in the levels of COL1A1 in the media compared with the vector control (Figure 2A). Interestingly, the HSP47 overexpression also significantly elevated intracellular levels of COL1A1 to 300% ± 15% compared with the vector control. Thus, our findings indicate that HSP47 overexpression in fibroblasts not only enhances the secretion of type I collagen but also increases the expression of type I collagen.

Furthermore, a conventional RT-PCR analysis showed an increase in both *COL1A1* and *COL1A2* mRNA levels upon HSP47 overexpression (Figure 2B, Left). A quantitative RT-PCR analysis revealed a significant upregulation of *COL1A1* mRNA and *COL1A2* mRNA levels to 177% ± 43.7% and 178% ± 42.4%, respectively, compared to the vector control (Figure 2B, Right). These results suggest that HSP47 overexpression increases the transcription of type I collagen genes, thereby promoting the synthesis and secretion of type I collagen in fibroblasts.

### 3.3. IRE1α Inhibitors Block the HSP47-Induced Increase in Type I Collagen Secretion and Expression in Human Fibroblasts

**Figure 3:**
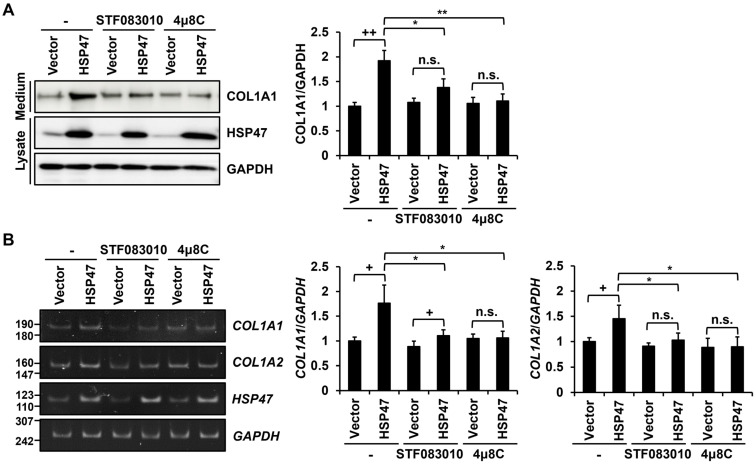
Effect of IRE1α RNase domain inhibitors on the secretion and expression of type I collagen in fibroblasts expressing HSP47. The IRE1α RNase domain inhibitors, STF083010 (40 μM) and 4μ8C (50 μM), were treated with serum-free medium for 24 h in foreskin fibroblasts infected with HSP47 lentivirus. (**A**) Protein levels of COL1A1, HSP47, and GAPDH were analyzed using Western blotting. The levels of type I collagen secretion were analyzed in the media and the levels HSP47 and GAPDH were analyzed in cell lysates. (**B**) mRNA levels of the *COL1A1*, *COL1A2*, *HSP47*, and *GAPDH* genes were analyzed by conventional RT-RCR. Relative protein and mRNA levels were normalized by GAPDH protein and mRNA levels. Each value in the graph represents the mean ± SD value of at least three independent experiments. * *p* < 0.05, ** *p* < 0.01 vs. HSP47 with no treatment; + *p* < 0.05, ++ *p* < 0.01 vs. vector virus infection. n.s.: not significant.

We subsequently investigated the mechanism by which HSP47 enhances the expression of type I collagen in human foreskin fibroblasts. It has been reported that HSP47 binds to the α isoform of IRE1 (IRE1α), leading to its activation [37]. Therefore, we examined whether IRE1α activation is involved in the HSP47-dependent induction of type I collagen. To address this, we treated human foreskin fibroblasts overexpressing HSP47 with the IRE1α RNase domain inhibitors, STF083010 and 4µ8C. HSP47 overexpression alone significantly increased COL1A1 secretion to 192% ± 20.8%. However, treatment with STF083010 and 4μ8C alongside HSP47 overexpression reduced type I collagen secretion to 138% ± 17.6% and 110% ± 14.1%, respectively (Figure 3A). Furthermore, HSP47 overexpression alone significantly increased the *COL1A1* and *COL1A2* mRNA levels to 176% ± 36.8% and 145% ± 26.4%, respectively. However, treatments with STF083010 and 4μ8C alongside HSP47 overexpression reduced the *COL1A1* mRNA levels to 111% ± 11.7% and 106% ± 13.5%, respectively, and reduced the *COL1A2* mRNA levels to 103% ± 14.0% and 89.7% ± 19.5%, respectively (Figure 3B). Based on these results, we posit that the HSP47-induced increase in the expression of type I collagen in human fibroblasts requires the RNase activity of IRE1α.

### 3.4. HSP47 Induces Splicing of XBP1 in Human Fibroblasts

**Figure 4:**
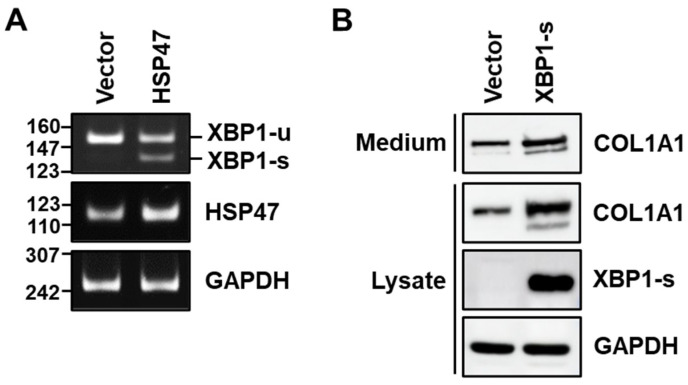
Effect of HSP47 expression on XBP1 splicing and the effect of XBP1-s expression on the secretion and expression of type I collagen in fibroblasts. HSP47 was overexpressed in human foreskin fibroblasts using HSP47 lentivirus. (**A**) Splicing of *XBP1* mRNA was analyzed by RT-PCR in fibroblasts at 24 h of incubation with complete media after viral infection. (**B**) XBP1-s was overexpressed in fibroblasts using XBP1-s lentivirus. At 24 h of incubation with complete medium after viral infection, the cells were incubated with serum-free medium for an additional 24 h. Media and cell lysates were subjected to 9% SDS-PAGE followed by Western blotting.

Given our observation that the inhibition of IRE1α RNase activity reduces HSP47-induced type I collagen expression, we proceeded to investigate whether the overexpression of HSP47 in human foreskin fibroblasts affects the splicing of *XBP1* RNA. Upon infecting fibroblasts with the HSP47 lentivirus, a PCR product derived from spliced *XBP1* (*XBP1-s*) mRNA, spliced from unspliced *XBP1* (*XBP1-u*) mRNA, was detected, while infection of the vector virus did not yield a PCR product derived of *XBP1-s* mRNA (Figure 4A).

Next, we explored whether XBP1-s is able to increase type I collagen in human foreskin fibroblasts. Following the infection of fibroblasts with XBP1-s lentivirus and a control vector lentivirus, comparable levels of GFP expression were observed in fibroblasts (Figure S2), indicating similar infection efficiencies. Overexpressing XBP1-s by infecting fibroblasts with XBP1-s lentivirus resulted in an increase not only in the secretion of COL1A1 into the medium but also in the intracellular level of COL1A1, mirroring the effects of HSP47 overexpression (Figure 4B). These findings affirm that IRE1α-induced XBP1 splicing plays a crucial role in HSP47-induced type I collagen expression in human fibroblasts.

### 3.5. Overexpression of HSP47 or XBP1-s Increases Nuclear Translocation of β-Catenin in Human Fibroblasts

**Figure 5:**
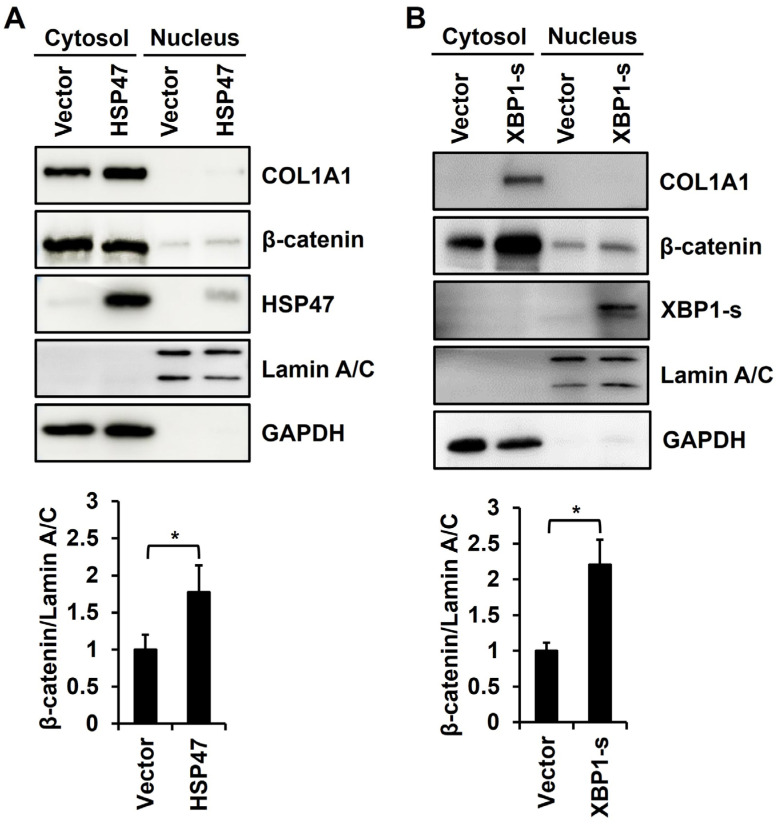
Effect of HSP47 or XBP1-s expression on nuclear localization of β-catenin in fibroblasts. HSP47 (**A**) and XBP1-s (**B**) were overexpressed in human foreskin fibroblasts using HSP47 or XBP1-s lentivirus. At 24 h of incubation with complete medium after viral infection, the cells were incubated with serum-free medium for an additional 24 h. The cell lysates were fractionated into nuclear and cytosolic fractions. Protein levels of COL1A1, β-catenin, HSP47, XBP1-s, Lamin A/C, and GAPDH were analyzed using Western blotting. Each value in the graph represents the mean ± SD value of nuclear β-catenin relative to Lamin A/C from three independent experiments. * *p* < 0.05 vs. vector virus infection.

It is known that XBP1-u decreases but XBP1-s increases the stability of β-catenin [38,39]. Therefore, we analyzed whether increased amounts of XBP1-s would enhance the stability and nuclear localization of β-catenin in human foreskin fibroblasts. The overexpression of HSP47, which induces XBP1 splicing and consequently increases XBP1-s levels, significantly augmented the nuclear translocation level of β-catenin in human fibroblasts to 177% ± 36.2% (Figure 5A). In addition, the overexpression of XBP1-s also showed a significant increase in the nuclear localization of β-catenin to 220% ± 35.2%, surpassing the effect observed with HSP47 overexpression (Figure 5B).

### 3.6. Overexpression of HSP47 or XBP1-s, as well as Treatment with Wnt3a, Enhances TCF Reporter Activity

**Figure 6:**
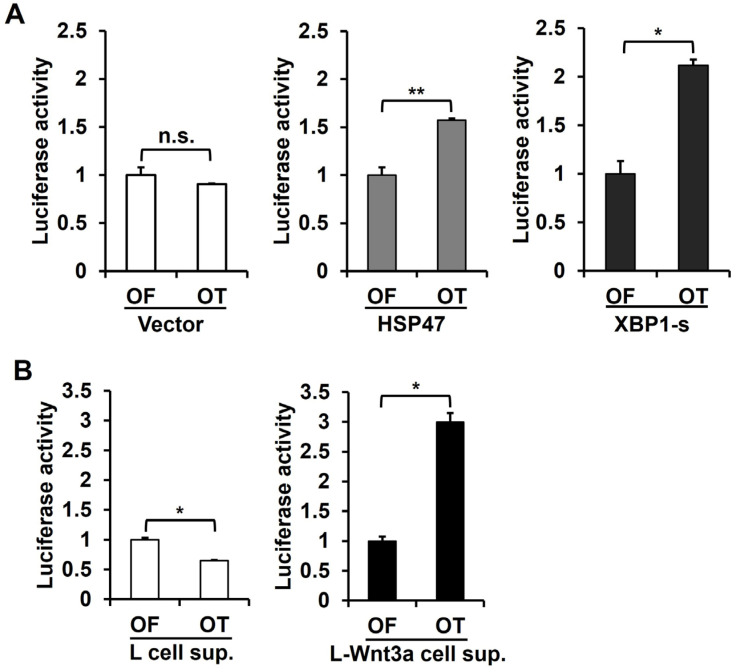
Effect of HSP47 or XBP1-s expression or Wnt3a treatment on β-catenin/TCF-dependent transcriptional activity in fibroblasts. (**A**) HSP47 or XBP1-s was overexpressed in human foreskin fibroblasts using HSP47 or XBP1-s lentivirus. At 24 h of incubation with complete media after viral infection, the cells were transfected with pGL3-OT (wild-type) or pGL3-OF (mutant) plasmid and then incubated in complete medium for 24 h. (**B**) Human foreskin fibroblasts were transfected with pGL3-OT or pGL3-OF and then treated with L-cell (L-cell sup.) or L-Wnt3a-cell (L-Wnt3a sup.) supernatant in complete medium for 24 h. Cell lysates were subjected to dual-luciferase reporter assay. Luciferase activity was quantified by calculating the ratio of firefly luciferase activity (560 nm) to *Renilla* luciferase activity (480 nm) and expressed as a relative value. The graphs show relative luciferase activity levels by the wild-type TCF reporter (OT) relative to the levels by the mutant TCF reporter (OF). Each value represents the mean ± SD of three independent experiments. * *p* < 0.05, ** *p* < 0.01 vs. pGL3-OF luciferase activity. n.s.: not significant.

To investigate whether the overexpression of HSP47 or XBP1-s can activate β-catenin/TCF target genes, we performed a luciferase reporter assay. T-cell factors (TCF) reporter plasmids, pGL3-OT (OT) containing wild-type TCF4-binding sites, and pGL3-OF (OF) containing mutant TCF-4 binding sites were transfected into human foreskin fibroblasts overexpressing HSP47 or XBP1-s. In fibroblasts infected with the vector virus, the luciferase activity of OT was comparable to that of OF. However, in fibroblasts overexpressing HSP47, the luciferase activity of OT increased to 157% ± 1.8% compared to that of OF. Similarly, in fibroblasts overexpressing XBP1-s, the luciferase activity of OT increased to 212% ± 5.8% compared to that of OF (Figure 6A).

Wnt3a is known to increase nuclear β-catenin levels in fibroblasts [40]. To demonstrate that TCF reporter activity is influenced by nuclear β-catenin, human foreskin fibroblasts transfected with OF or OT were treated with the conditioned medium of L cells overexpressing Wnt3a (L-Wnt3a) as a source of Wnt3a and the conditioned medium of L cells as a control. Fibroblasts treated with L-Wnt3a cell supernatant exhibited an increase in the luciferase activity of OT to 300% ± 15.0% compared to that of OF. In contrast, treatment of the L-cell supernatant decreased the luciferase activity of OT to 65% ± 0.9% compared to OF (Figure 6B). These results support the notion that the elevated expression of HSP47 or XBP1-s enhances the activity of the β-catenin/TCF bipartite transcription factor.

### 3.7. Treatment of Wnt3a Increases Nuclear Translocation of β-Catenin and Expression of Type I Collagen in Human Fibroblasts

**Figure 7:**
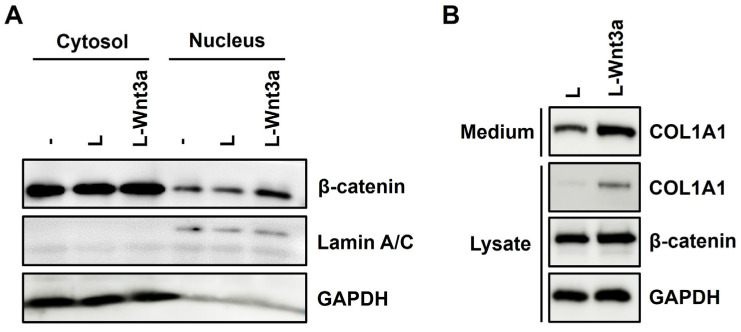
Effect of Wnt3a treatment on nuclear localization of β-catenin and expression of type I collagen in fibroblasts. (**A**) Human foreskin fibroblasts were treated in serum-free medium with L-Wnt3a-cell supernatant (L-Wnt3a) or L-cell supernatant (L) and were harvested after 24 h. The cell lysates were fractionated into nuclear and cytosolic fractions and were subjected to 9% SDS-PAGE. Protein levels of β-catenin, Lamin A/C, and GAPDH were analyzed in the fractions. (**B**) Human foreskin fibroblasts were treated in serum-free medium with L-Wnt3a-cell supernatant (L-Wnt3a) or L-cell supernatant (L). The cells were harvested after 48 h of treatment. The levels of type I collagen secretion were analyzed in the media and the levels type I collagen, β-catenin, and GAPDH were analyzed in cell lysates.

Subsequently, we investigated whether the increased nuclear β-catenin levels could enhance type I collagen secretion in human foreskin fibroblasts. Following the treatment of conditioned medium from L-Wnt3a cells to fibroblasts, there was an increase in β-catenin levels in the nuclear fraction compared to the treatment with conditioned medium from L cells (Figure 7A). Treatment with the conditioned medium from the L-Wnt3a cells resulted in elevated levels of COL1A1 in both the medium and cell lysate of fibroblasts (Figure 7B). These results demonstrate that Wnt3a increases the secretion and expression of type I collagen. Collectively, our findings support the conclusion that overexpression of HSP47 or XBP1-s can enhance the expression of type I collagen by increasing the nuclear translocation of β-catenin.

## 4. Discussion

Decreased collagen levels lead to loss of skin elasticity, resulting in the formation of wrinkles and sagging skin [41,42]. Collagen plays a significant role in maintaining the youthful appearance and health of the skin. We observed a decrease in mRNA and protein levels of HSP47 in the skin tissues of old mice (24 months old) compared to young mice (3 months old). Since HSP47 is known to play a role in collagen folding and assembly, we hypothesized that the observed decrease in HSP47 expression in skin aging would lead to a reduction in collagen levels in the skin.

We investigated the impact of increased HSP47 expression on type I collagen secretion in human foreskin fibroblasts. The overexpression of HSP47 in human fibroblasts through lentivirus led to a notable augmentation in type I collagen secretion. Surprisingly, heightened HSP47 expression led to an increase in intracellular levels of type I collagen, contrary to the anticipated decrease if HSP47 merely facilitated folding and assembly. Moreover, the overexpression of HSP47 demonstrated an elevation in *COL1A1* and *COL1A2* mRNA levels within the fibroblasts, supporting the notion that HSP47 functions not only as a collagen chaperone but also as a primary stimulator for collagen type I production. Previous studies have indicated a correlation between HSP47 levels in the ER and both intracellular and extracellular levels of type I collagen assembly in smooth muscle cells (SMCs) [43]. Furthermore, the upregulation of HSP47 has been shown to increase mRNA expression levels of collagen types I, III, and V in scleral fibroblasts [44]. Consequently, our discovery that HSP47 induces the expression of type I collagen in fibroblasts aligns with and reinforces these prior findings.

While some studies have suggested the potential for HSP47 to upregulate collagen expression [44], the specific signaling pathways responsible for this effect remain unclear. Thus, we sought to elucidate the regulatory mechanisms through which HSP47 influences type I collagen expression. Previous interactome screening of IRE1α regulators revealed that HSP47 forms a high-affinity direct binding with the ER luminal domain of IRE1α in vitro. This interaction displaces the negative regulator BiP from the complex, facilitating IRE1α oligomerization. Consequently, HSP47 expression enhances IRE1α activation, amplifying downstream signaling and effectively mitigating ER stress [6,37]. Given this information, we hypothesized that IRE1α might play a role in the upregulation of type I collagen mediated by HSP47. To test this hypothesis, we treated foreskin fibroblasts overexpressing HSP47 with RNase domain inhibitors of IRE1α, STF083010 and 4μ8C, and assessed changes in type I collagen expression. The use of these inhibitors resulted in a decrease in the secretion and expression of type I collagen, which had been increased by HSP47. Based on these findings, we conclude that the activity of the IRE1α RNase domain is a critical factor in the upregulation of type I collagen expression induced by HSP47.

The activation of the RNase domain of IRE1α initiates the unconventional splicing of the XBP1 transcript, influencing the expression of ER components responsive to ER stress [45]. In this context, we investigated whether the splicing of XBP1 is implicated in HSP47-induced type I collagen expression in foreskin fibroblasts. As anticipated, the splicing of XBP1 transcripts from *XBP1-u* mRNA to *XBP1-s* mRNA increased in fibroblasts with heightened expression of HSP47. Furthermore, the overexpression of XBP1-s also resulted in increased type I collagen secretion and expression. Collectively, these findings confirm that HSP47 has the capability to enhance the activation of IRE1α, leading to the splicing of XBP1 and subsequently augmenting type I collagen expression.

We then investigated how XBP1-s, induced by the increased expression of HSP47, enhances type I collagen expression in human dermal fibroblasts. A study revealed that heightened binding of HSP47 to IRE1α under high glucose conditions in Müller cells leads to an increase in XBP1-s and HIF-1α expression [46]. Keloid fibroblasts exhibited higher levels of HIF-1α than normal dermal fibroblasts and also showed an increase in both intracellular and extracellular COL1A1 levels under hypoxic conditions, resulting in the stabilization and accumulation of HIF-1α [47]. Thus, it is conceivable that HSP47 amplifies type I collagen synthesis via the IRE1α/XBP1-s/HIF-1α pathway. However, because we did not analyze fibroblasts under hyperglycemic or hypoxic conditions, we focused on the possibility that XBP1-s regulates β-catenin activity. XBP1 is known to play two different roles in increasing the stability of β-catenin. Firstly, the degradation of β-catenin is enhanced by XBP1-u. XBP1-u possesses a C-terminal degradation domain that promotes the ubiquitin–proteasomal degradation of β-catenin and inhibits its total and nuclear accumulation [38,48]. Upon the activation of IRE1α in response to ER stress or other triggers, XBP1-u is converted to XBP1-s [49,50], leading to a reduction in XBP1-u and facilitating the accumulation of β-catenin. Secondly, XBP1-s is also known to enhance the nuclear translocation of β-catenin. In vascular endothelial cells, the overexpression of XBP1-s increases the phosphorylation of glycogen synthase kinase 3β (GSK3β) within the degradation complex, suppresses GSK3β activity, dissociates β-catenin from phospho-GSK3β, and increases its nuclear translocation [39]. Given these findings, a decrease in XBP1-u and an increase in XBP1-s were expected to promote the nuclear translocation of β-catenin. As expected, we observed that HSP47 expression increased nuclear β-catenin levels, XBP1-s expression elevated both cytosolic and nuclear β-catenin levels, and HSP47 or XBP1-s expression increased the secretion of type I collagen. Furthermore, we demonstrated that HSP47 or XBP1-s expression increased β-catenin/TCF-driven luciferase activity.

It has been known that Wnt/β-catenin signaling is one of the signaling pathways that increase the expression of collagens [51]. Nuclear β-catenin interacts with TCF and lymphoid enhancer factors (TCF/LEF) to activate and/or repress transcription of its target genes [52,53]. One of the target genes, *COL1A1*, has four TCF binding sites at the promoter region: nucleotide positions -29 to -25, -484 to -480, -1642 to -1638, -2713 to -2709 [54]. β-catenin overexpression increased the expression of type I and III collagens in immortalized fibroblasts. Although TCF binding sites in the *COL1A2* promoter have not been defined yet, increasing amounts of β-catenin resulted in a dose-dependent increase in *COL1A2* luciferase reporter activity [55]. Consistent with these findings, we also detected increased expression and secretion of type I collagen upon treatment of the Wnt3a-expressing L-cell supernatant.

**Figure 8:**
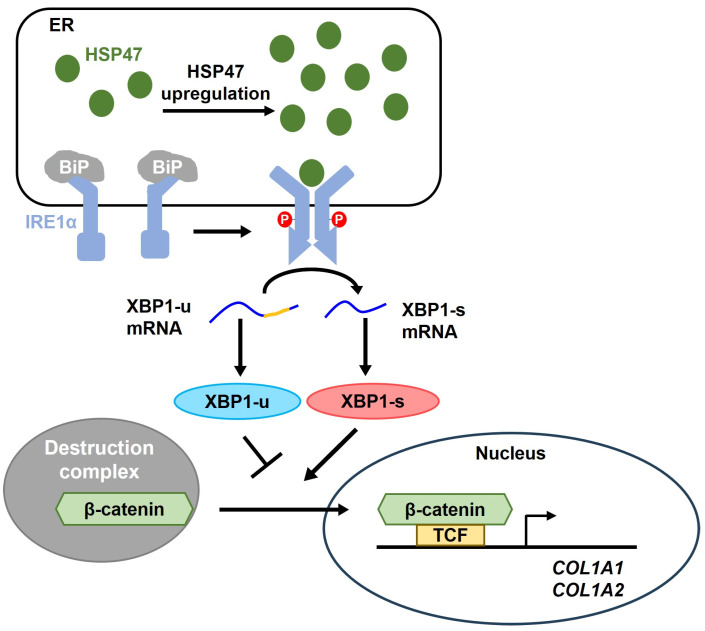
Schematic representation of the proposed mechanism of HSP47-mediated increase in type I collagen expression. When HSP47 is increased due to ER stress or other reasons in the ER, it can displace BiP that was originally bound to IRE1α. Subsequently, HSP47 facilitates the activation of IRE1α, enhances the splicing of XBP1, and thus decreases XBP1-u levels while increasing XBP1-s levels. Since the stability of β-catenin is known to be decreased by XBP1-u but increased by XBP1-s, the HSP47-induced activation of IRE1α results in an increase in nuclear levels of β-catenin, activating the β-catenin/TCF transcription factor and thereby upregulating the expression of the target genes, such as *COL1A1* and *COL1A2*.

Our findings propose a novel mechanism wherein the expression of HSP47 activates IRE1α, leading to the induction of XBP1 splicing. This activation subsequently enhances the nuclear translocation of β-catenin, resulting in elevated collagen synthesis in dermal fibroblasts (Figure 8). This novel mechanism complements the traditionally acknowledged role of HSP47 in facilitating the folding and assembly of collagens. Given the observed reduction in HSP47 during skin aging, augmenting HSP47 expression could potentially serve as a beneficial strategy to decelerate the aging process of the skin. Furthermore, our research holds promise in contributing to the regulation of collagen homeostasis, potentially offering insights into the treatment of diseases resulting from collagen deficiency, such as osteoporosis.

## 5. Conclusions

Our study elucidates the crucial role of HSP47 in modulating collagen homeostasis, emphasizing its significance as an inducer of type I collagen expression, in addition to its well-established collagen-specific chaperone function. The observed decrease in *HSP47* mRNA levels in the skin of aged mice highlights its potential involvement in age-related alterations. Particularly, our study shows that increased *HSP47* mRNA levels positively influence the expression and secretion of type I collagen in fibroblasts. Moreover, our mechanistic findings reveal the complex interplay involving IRE1α RNase activity, *XBP1* mRNA splicing, and the nuclear translocation of β-catenin orchestrated by HSP47. These pathways collectively contribute to the enhanced expression of type I collagen. The identified regulatory mechanisms offer promising opportunities for strategies to control collagen dynamics, suggesting that interventions targeting HSP47-mediated processes, particularly through modulating nuclear β-catenin levels via IRE1α activation and XBP1 splicing, have potential therapeutic value.

## Data Availability

Data are included within the article and Supplementary Material.

## Supplementary Materials

The following supporting information can be downloaded at: https://www.mdpi.com/article/10.3390/cells13060527/s1, Table S1: Primer sequences used for reverse transcription-polymerase chain reaction of *COL1A1, COL1A2, HSP47, XBP1*, and *GAPDH* mRNAs; Figure S1: Comparison of the infection efficiencies of vector and HSP47 viruses in fibroblasts; Figure S2: Comparison of the infection efficiencies of vector and XBP1-s viruses in fibroblasts.
