# Supplementary material for: HSP47 Increases the Expression of Type I Collagen in Fibroblasts through IRE1α Activation, XBP1 Splicing, and Nuclear Translocation of β-Catenin
Source: Cells. 2024 Mar 17;13(6):527. doi: 10.3390/cells13060527 (PMC10969015; doi:10.3390/cells13060527)
Supplement: Supplementary file 1 [file cells-13-00527-s001.zip › cells-2801017-supplementary.pdf]

Supplementary materials

HSP47 increases the expression of type I collagen in fibroblasts through IRE1α activation, XBP1 splicing, and nuclear translocation of β-catenin

So Young Ham [1], Min Ju Pyo [1], Moonkyung Kang[2], Yeon-Soo Kim [2,3], Dong Hun Lee [4,5,6], Jin Ho Chung [4,5,6,7], and Seung-Taek Lee [1,*]

1 Department of Biochemistry, College of Life Science and Biotechnology, Yonsei University, Seoul 03722, Republic of Korea; soyoung@yonsei.ac.kr (S.Y.H.); vyalswn2525@naver.com (M.J.P.)
2 R&D Center, artiCure Inc., Daejeon 34134, Republic of Korea; moonkkang73@icgm.kr (M.K.); kimys58@cnu.ac.kr (Y.-S.K.)
3 Graduate School of New Drug Discovery and Development, Chungnam National University, Daejeon 34134, Republic of Korea
4 Department of Dermatology, Seoul National University College of Medicine, Seoul 03080, Republic of Korea; ivymed27@snu.ac.kr (D.H.L.); jhchung@snu.ac.kr (J.H.C.)
5 Laboratory of Cutaneous Aging Research, Biomedical Research Institute, Seoul National University Hospital, Seoul 03080, Republic of Korea
6 Institute of Human-Environment Interface Biology, Seoul National University, Seoul 03080, Republic of Korea
7 Institute on Aging, Seoul National University, Seoul 03080, Republic of Korea
* Correspondence: stlee@yonsei.ac.kr; Tel.: +82-2-2123-2703

Table S1. Primer sequences used for reverse transcription-polymerase chain reaction of *COL1A1, COL1A2, HSP47, XBP1,* and *GAPDH* mRNAs.

Gene symbol	Nucleotide sequence	Nucleotide position	Annealing temp. (°C)	GenBank #
COL1A1-F	5′-ACAGCGTCACTGTCGATGGCTG-3′	4341–4372	55	NM_000088
COL1A1-R	5′-GGAGGGAGTTTACAGGAAGCAGACAG-3′	4522–4497		
COL1A2-F	5′-GAGGGCAACAGCAGGTTCACTTACAC-3′	4044-4069	55	NM_000089
COL1A2-R	5′-GTCAGCACCACCGATGTCCAAAG-3′	4196-4174		
HSP47-F	5′-AAGCTGTTCTACGCCGACCACC-3′	1227-1248	56	NM_001235
HSP47-R	5′- CGTCTCGCATCTTGTCACCCTTAG -3′	1338-1315		
XBP1-F	5′- GAGAACCAGGAGTTAAGACAGCGCT-3′	429-444	62	NM_001079539
XBP1-R	5′- GTGACAACTGGGCCTGCACCT-3′	563–543		
GAPDH-F	5′-ACTGCTTAGCACCCCTGGCCA-3′	488–508	57	BC023632
GAPDH-R	5′-TTGGCAGTGGGGACACGGAAG-3′	740–720		

F: forward primer and R: reverse primer

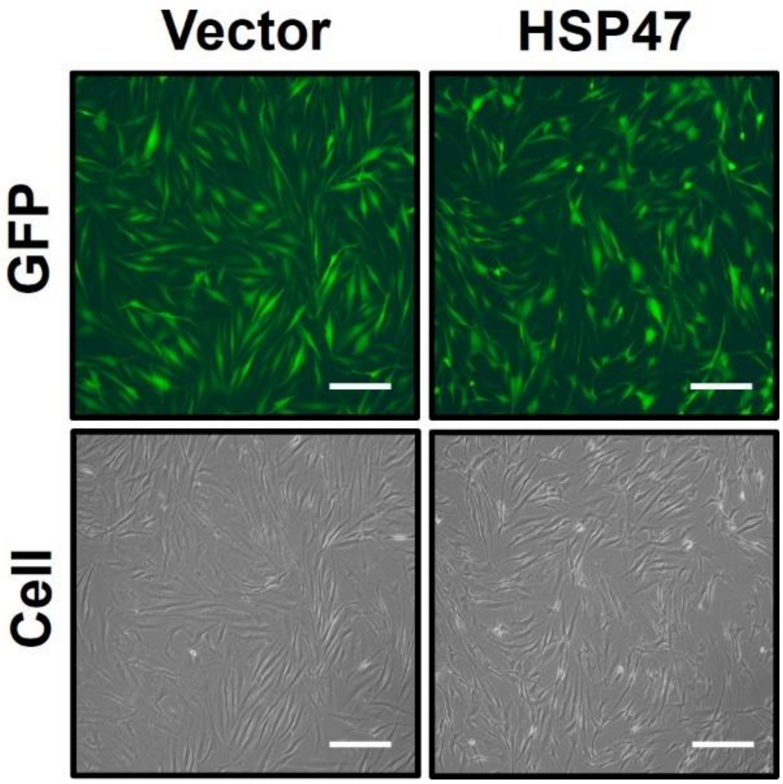

Figure S1. Comparison of the infection efficiencies of vector and HSP47 viruses in fibroblasts. Human foreskin fibroblasts were infected with Vector or HSP47 lentiviruses coexpressing GFP. After 24-h incubation in complete medium followed by 24-h incubation in serum-free medium, fibroblasts were analyzed to monitor the infection efficiency using fluorescence and phase contrast microscopy. Magnification: ×100. Bar=200 μm.

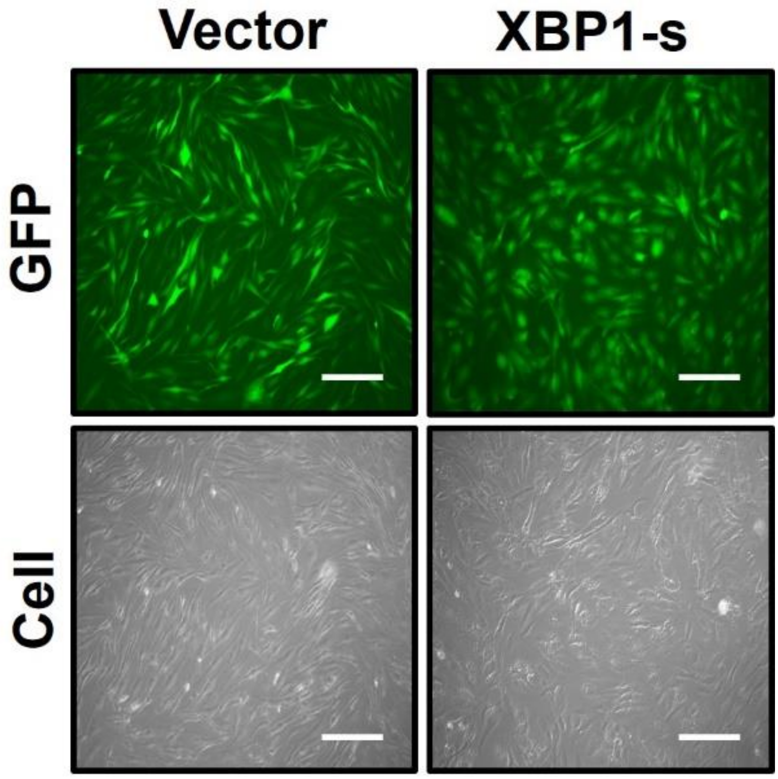

Figure S2. Comparison of the infection efficiencies of vector and XBP1-s viruses in fibroblasts. Human foreskin fibroblasts were infected with Vector or XBP1-s lentiviruses coexpressing GFP. After 24-h incubation in complete medium followed by 24-h incubation in serum-free medium, fibroblasts were analyzed to monitor the infection efficiency using fluorescence and phase contrast microscopy. Magnification: ×100. Bar=200 μm.
